# First person – Joshua Hack

**DOI:** 10.1242/bio.060410

**Published:** 2024-04-24

## Abstract

First Person is a series of interviews with the first authors of a selection of papers published in Biology Open, helping researchers promote themselves alongside their papers. Joshua Hack is first author on ‘ Machine learning models reveal distinct disease subgroups and improve diagnostic and prognostic accuracy for individuals with pathogenic *SCN8A* gain-of-function variants’, published in BiO. Joshua is a Data Scientist in the lab of Dr Michael Hammer at the University of Arizona, investigating the mechanisms of diseases and expanding the phenotypic landscapes of rare genetic diseases towards the goal of improving treatment strategies on an individual basis.


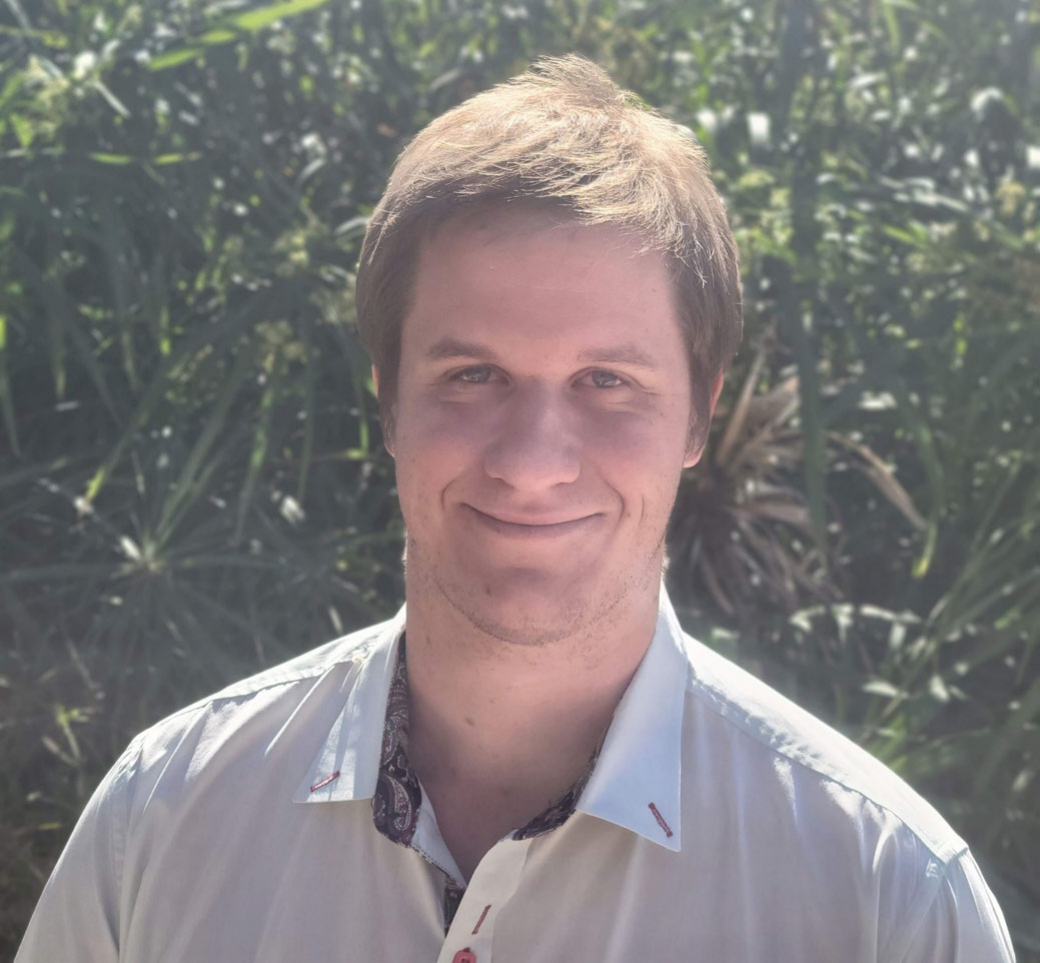


**Joshua Hack**

**Describe your scientific journey and your current research focus**

I've always been interested in understanding the underlying cause of phenomena that are present around me. Naturally, this led me to develop a passion for science, particularly genetics applied in medicine and evolutionary biology. My initial research focus was autoimmune diseases, which have impacted many of my family members including myself, but when COVID-19 began, the lab I was working in was unable to support my research. It was then that I switched to my current position studying a family of rare diseases caused by mutations in the SCN8A gene and went from a bench scientist to a computational biologist specializing in machine learning. Over the last 4 years, I have enjoyed learning the various techniques for applying machine learning to patient databases and have further developed my research goals and interests for the future. While I eventually would like to return to the field of immunology and continue studying autoimmune diseases, rare disease research will always be a component of my career, as a way to serve communities that are often under-represented in translational research. My time working with SCN8A-related disorders has also instilled in me a passion for bioinformatics and in the future I will be leveraging both bench and computational methods to understand evolutionary origins and underlying mechanisms of the diseases and pathways I study.

**Who or what inspired you to become a scientist?**

There are a few researchers and philosophers that have had a profound impact on my desire to become a scientist, particularly Dr Hugh Ross, Dr Fazale Rana, and philosopher Kenneth Samples. I have been blessed enough to be able to foster personal relationships with these scholars and continue to learn from them. These three men have played a significant role in shaping my understanding of science and how I think about the natural world I study. More than that, these men have inspired me in how they communicate science and complex topics to those not involved in their fields, and are a large reason as to why I care so deeply about not just research for education and dissemination of breakthrough findings. Their mentorship has also emphasized the importance of a curious mind and pursuing knowledge and insight from many fields, and not restricting my studies to just genetics.

This paper uses a combination of typical machine learning approaches in the pursuit of building a tool that can help clinicians more accurately predict the progression of the disease SCN8A Epilepsy.

**How would you explain the main finding of your paper?**

This paper uses a combination of typical machine learning approaches in the pursuit of building a tool that can help clinicians more accurately predict the progression of the disease SCN8A Epilepsy. We use two approaches, one informed by clinical consensus (supervised) and one where the computational algorithm groups individuals together (unsupervised), to form three groups within a subset of the patient population. From there, we construct a total of four machine learning models that consider a patient's clinical presentation (i.e. seizure profile and developmental delays) and produce a decision on which group that individual is likely in. These models perform with high performance on features that are easily assessed in clinical settings early in the disease progression. An unexpected finding was disagreement between the approaches in what differentiated each of the clinical subgroups. The supervised approach results in a mild to severe spectrum while the unsupervised approach seems to have three categories that are partially ordered (that is, 1A versus 1B versus 2). This discordance results in the proposal of a hypothesis that developmental and epileptic encephalopathies may be separable into subgroups where either developmental disability or epilepsy are the dominant component of the disease.

**What are the potential implications of this finding for your field of research?**

This study succeeds in building a machine learning tool that can aid in clinical decision making for prognosis of a rare disease. The tool constructed here may be extremely helpful for characterizing patients and providing a more specific and realistic expected progression of the disease for caregivers and patients. Machine learning tools like this are greatly beneficial in rare disease as clinicians will rarely treat more than 10 individuals with a specific disease.

This study also proposes a hypothesis that may shift the understanding of the phenotypic landscape of SCN8A-related diseases. Further testing on the hypothesis proposed here may support the idea that genetic developmental and epileptic encephalopathies are able to be further differentiated into more specific subgroups.

**Figure:**
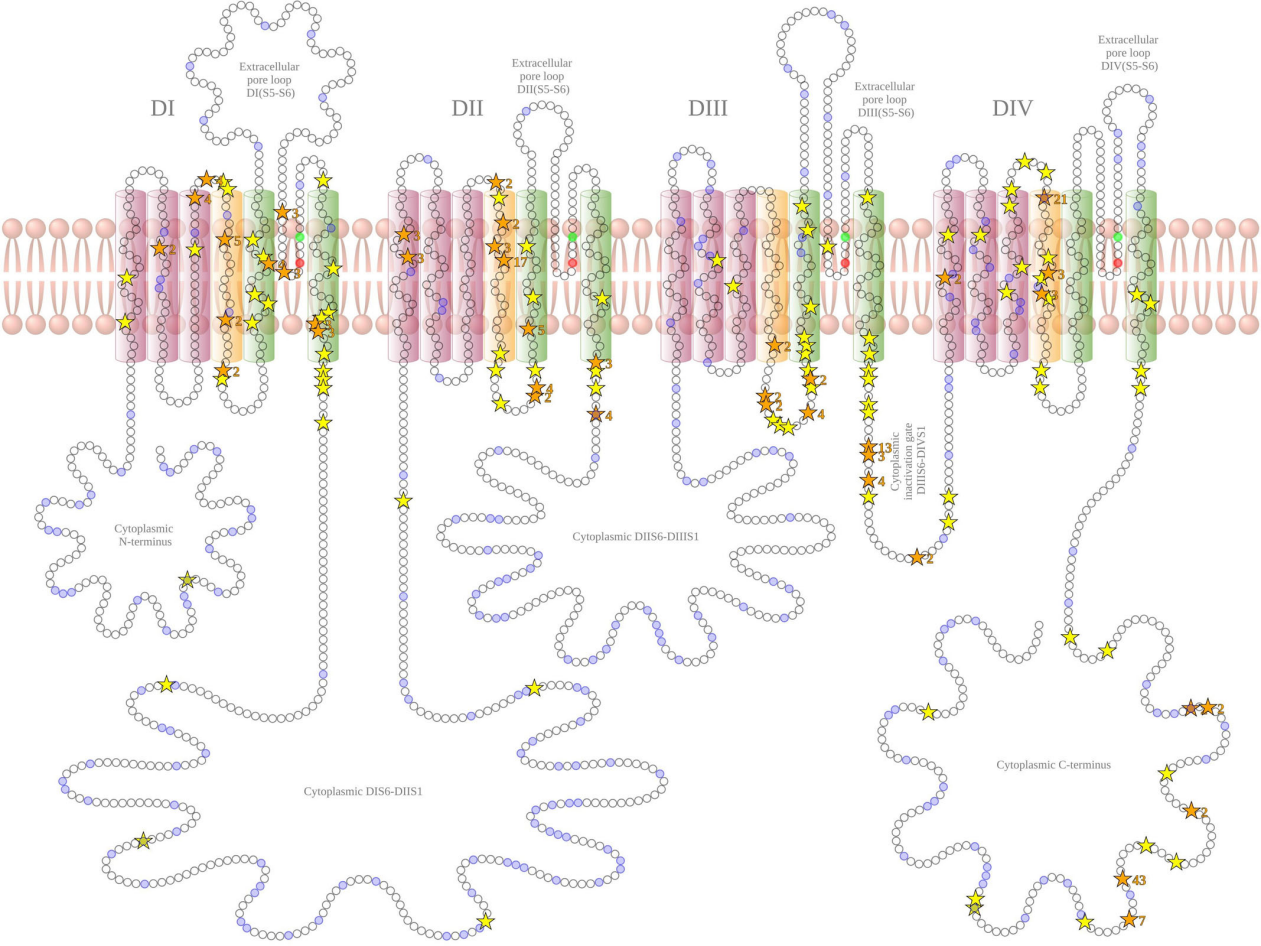
**Reported SCN8A variants associated with gain of function features and their location in the voltage gated sodium channel NaV1.6 as of May 2023.** Yellow stars indicate single instances of variants at the associated amino acid position while orange stars indicate multiple instances of variants and are marked with the number of reports.

**Which part of this research project was the most rewarding?**

I had the opportunity to present this study and a previous study with similar goals at a gathering of families, clinicians, and researchers involved with SCN8A. The time I spent with the caregivers and affected individuals was incredibly encouraging to me and helped me re-orient my perspective. Their gratitude and excitement about all of the projects that were presented was refreshing and had a profound impact on me. There is nothing I could say that would capture the extent of my gratitude to this disease community and how they consistently encourage, engage, and challenge us as researchers and clinicians to pursue challenging questions.

**What do you enjoy most about being an early-career researcher?**

I enjoy the dynamic that I have with other researchers further along in their careers. It's wonderful being able to just sit down with others and hear about their own experiences, advice, mistakes, and successes while being considered on equal footing with them. The willingness and excitement that they have to mentor me and share their stories with are incredible for my own development and way of thinking.

**What piece of advice would you give to the next generation of researchers?**

My biggest piece of advice is don't be afraid to challenge conventional knowledge and consensus. While many times, field consensus is well supported, it's important to understand that science is constantly progressing and what may have been consensus may not be in the coming decades. The greatest scientific achievements have always been a challenge to conventional knowledge, and it's vital that we never restrict ourselves to a confined box. My second piece of advice is to bask in the wonder of the universe you study. We so often take for granted how incredible the things we study are and it's vital that we as researchers maintain the wonder we had as young researchers. I've found that the best vaccine against disillusionment and burnout is a mindset of wonder.

I've found that the best vaccine against disillusionment and burnout is a mindset of wonder.

**What's next for you?**

I am beginning a PhD program in genetics in the fall of 2024, program pending. I hope to continue contributing my time and expertise to the SCN8A community in some capacity while I pursue a PhD. My interests are primarily in genetic mechanisms of autoimmunity, evolutionary origins of immunological pathways, and rare diseases, particularly those resistant to therapy. I also hope to expand my research to look at coral bleaching through the lens of immunology as a way to incorporate one of my hobbies (SCUBA Diving) into my research.
